# Topology of brain functional connectivity networks in posttraumatic stress disorder

**DOI:** 10.1016/j.dib.2018.08.198

**Published:** 2018-09-06

**Authors:** Teddy J. Akiki, Christopher L. Averill, Kristen M. Wrocklage, J. Cobb Scott, Lynnette A. Averill, Brian Schweinsburg, Aaron Alexander-Bloch, Brenda Martini, Steven M. Southwick, John H. Krystal, Chadi G. Abdallah

**Affiliations:** a National Center for PTSD – Clinical Neurosciences Division, US Department of Veterans Affairs, West Haven, CT, United States; b Department of Psychiatry, Yale University School of Medicine, New Haven, CT, United States; c Gaylord Specialty Healthcare, Department of Psychology, Wallingford, CT, United States; d Department of Psychiatry, Perelman School of Medicine, University of Pennsylvania, Philadelphia, PA, United States; e VISN4 Mental Illness Research, Education, and Clinical Center at the Philadelphia VA Medical Center, Philadelphia, PA, United States

**Keywords:** fMRI, PTSD, Functional connectivity, Graph theory, Veterans, Intrinsic connectivity networks, Default mode network

## Abstract

Here we present functional neuroimaging-based network data (focused on the default mode network) collected from a cohort of US Veterans with history of combat exposure, combined with clinical assessments for PTSD and other psychiatric comorbidities. The data has been processed and analyzed using several network construction methods (signed, thresholded, normalized to phase-randomized and rewired surrogates, functional and multimodal parcellation). An interpretation and discussion of the data can be found in the main NeuroImage article by Akiki et al. [51]

**Specifications table**

**Table:** 

Subject area	*Psychiatry*
More specific subject area	*Neuroimaging, Posttraumatic Stress Disorder*
Type of data	*Tables, graphs, figures, text*
How data was acquired	*Siemens TIM Trio 3.0T magnet (32-channel head coil): Structural and functional MRI; clinical assessments*
Data format	*Analyzed*
Experimental factors	*Resting state acquisitions*
Experimental features	*US Veterans with history of combat exposure were assessed for PTSD symptoms and underwent MRI scans*
Data source location	*West Haven, Connecticut, USA*
Data accessibility	*Analyzed results are included*

**Value of the data**

• Robustness of neuroimaging graph theory measures were assessed in a real-world sample of the combat-exposed population and can inform future studies.
• The default mode network was thoroughly examined in PTSD.
• The default mode network findings in PTSD can be compared to other disorders to further assess its utility as a biomarker.
• The variability due to different network construction methods from fMRI can be assessed.
• The effect of psychiatric comorbidities on brain network metrics can be assessed.

## 1 Data

### 1.1 Intro

Using a network-restricted approach graph theoretical approach, we found and reported evidence of altered functional connectivity within the default mode network (DMN) in posttraumatic stress disorder (PTSD). Briefly, overall connectivity strength (*S*) and global efficiency (*E*) were found to be negatively correlated with PTSD symptom severity, while the overall clustering coefficient was positively correlated with PTSD symptom severity (see main article in NeuroImage [51]). Here we provide additional data, including details of clinical assessments and robustness analyses using alternate processing methods.

### 1.2 Justification for using a dimensional approach

To date, the PTSD literature has largely used a binary diagnostic approach comparing patients with DSM diagnosis of PTSD to control groups with and without trauma history; often excluding subjects with subthreshold PTSD. A strength of this approach is the creation of large contrast between groups. However, it also creates a potentially artificial dichotomization, especially if trauma-related pathophysiological effects are on a continuum of biological abnormalities and clinical severity. In addition, the extent of subthreshold PTSD pathophysiology is often missed. A dimensional approach based on PTSD symptom severity in a trauma exposed population regardless of diagnosis may potentially better map to underlying circuitry alterations. It will also maintain high clinical relevance to Veterans suffering from PTSD symptoms without necessarily meeting all DSM criteria. As a continuous measure of symptom severity, we adopted the Clinician Administered PTSD Scale for the DSM-IV (CAPS) [1], which is a structured standardized interview and has been demonstrated to have a highly robust validity and inter-rater reliability [1], [2], [3].

### 1.3 Justification for selecting the default mode network

The DMN is highly relevant for disease states [4], [5], [6], [7], [8], [9]. Further, unlike other ICNs, the DMN is active at rest during internally-focused tasks and suppressed during goal-directed tasks [6], [9]. This makes the DMN a prime target for biomarker development, since the resting-state functional paradigm is convenient to recreate, and likely to be consistent, across studies. Despite its apparent importance, the DMN has not been fully investigated in PTSD, where previous studies were mostly limited to a small number of seeds. In this study, our primary aim was to establish a DMN-restricted approach that will systemically investigate the DMN-specific network characteristics in relation to PTSD symptoms.

### 1.4 Introduction to graph theory in neuroimaging

The emergence of complex network analyses of brain connectivity has sparked an interest in trying to explain psychopathology in terms of neuronal network dysfunction [5], [10]. In graph theory terms, each ROI is referred to as a node, and each connection (functional or anatomical) is referred to as an edge. In functional networks, edge weights often represent magnitudes of correlations. Networks are then constructed from these basic units, and numerous metrics can be used to describe their configuration [11].

One commonly used nodal metric is known as nodal strength, which consists of the sum of all neighboring edge weights (analogous to functional connectivity strength from the seed-based literature). Nodal strength can also be averaged across the whole network to characterize the overall within-network connectivity strength, or wiring investment (here referred to as *S*) [12].

Beyond connectivity strength, the brain develops under environmental pressure to maximize computational power given the restricted available resources; and it is theorized that an optimal level of integration—efficiency of information transfer across the network; and segregation—ability for specialized processing to occur within a highly interconnected region, is crucial in maintaining cost-effective and efficient information processing in the brain [12], [13], [14]. Indeed, a disrupted pattern of integration and segregation has been described in numerous psychopathologies. To index network integration, a graph measure known as global efficiency can be calculated directly at the level of the whole network, which measures efficiency of overall network communication across the network [12], [15], [16]. A common way to index network segregation is by calculating a nodal metric known as the clustering coefficient, which measures the tendency of nodes to cluster together [17], [18]. Like the nodal strength, the nodal clustering coefficient can be averaged across the network to characterize whole-network segregation (see [12] for a review of graph theory in neuroimaging).

## 2 Experimental design, materials and methods

### 2.1 Participants and clinical assessments

Full description of the study sample and assessments were previously reported [19], [20], [21]. Briefly, 65 combat-exposed US Veterans with successful scans were included in this study. Inclusion criteria required at least one combat deployment. Exclusion criteria included: psychotic disorder or bipolar disorder, attention-deficit/hyperactivity disorder, learning disorder, moderate or severe traumatic brain injury (TBI), brain tumor, epilepsy or other neurological disorders, current benzodiazepine use, and MRI contraindication. Depression, anxiety, and substance/alcohol use disorders as well as stable antidepressant regimens were not considered as basis for exclusion in order to improve external validity and generalizability of the findings to the target population. We employed a single-group dimensional approach to capture a continuous spectrum of PTSD symptoms.

The Clinician Administered PTSD Scale for the DSM-IV (CAPS) was used to assess PTSD diagnosis and symptom severity [1]. The Combat Exposure Scale (CES) was used to assess combat exposure [22]. The Structured Clinical Interview for the DSM-IV (SCID-IV) was used to assess psychiatric comorbidities [23]. The Beck Depression Inventory (BDI) and Beck Anxiety Inventory (BAI) were used to assess depressive and anxiety symptoms, respectively [24], [25]. The Wechsler Test of Adult Reading (WTAR) was used to estimate pre-exposure/pre-morbid intellectual functioning [26]. Sample characteristics are presented in Table 1.

**Table 1:** Demographic and clinical characteristics.

	**Mean ±*****SEM*****or %**
**N**	65
**Age (years)**	34.8 ± 1.2
**Sex (% female)**	11%
**WTAR Standard Score**	103.4 ± 1.0
**Education (years)**	14.0 ± 0.2
**CAPS**	43.4 ± 3.7
**CES**	17.8 ± 1.3
**BDI**	19.1 ± 1.5
**BAI**	13.1 ± 1.3
**DSM-IV Axis I**	69%
**PTSD**	54%
**MDD**	20%
**SUD**	20%
**Anxiety Disorder**	7%
**Psychotropic Medication**	37%
**Mild TBI**	63%
**Handedness (% left handed)**	17%

Abbreviations – SEM: Standard Error of Means; WTAR: Wechsler Test of Adult Reading; CAPS: Clinician Administered PTSD Scale for the DSM-IV; CES: Combat Exposure Scale; BDI: Beck Depression Inventory; BAI: Beck Anxiety Inventory; PTSD: Posttraumatic Stress Disorder; MDD: Major Depressive Disorder; SUD: Substance/Alcohol Use Disorder; Anxiety: Panic Disorder, Generalized Anxiety Disorder, Obsessive Compulsive Disorder; TBI: Traumatic Brain Injury.

### 2.2 Neuroimaging acquisition and processing

Details relevant to neuroimaging acquisition and processing have been reported in the main NeuroImage manuscript [51], but a copy is made available in Box 1 for the convenience of the reader.

Box 1 **Neuroimaging acquisition and processing. Adapted from the main article in NeuroImage [51].**

1. Neuroimaging Acquisition
  Imaging data were collected using a Siemens TIM Trio 3.0 T magnet with a 32-channel head coil. Three high-resolution structural MRI (sMRI) scans were used to improve surface delineation and enable subject-specific coregistration: 2 × T1-weighted MPRAGE (voxel size = 1 × 1 × 1 mm; TR = 2530 ms; TE = 2.71 ms; Flip = 7°); 1 × T2-weighted (voxel size = 1 × 1 × 1 mm; TR = 3200 ms; TE = 419 ms; Flip = 120°). Whole-brain functional data were acquired using two 5-min T2*-weighted BOLD resting-state runs (voxel size = 3.4 × 3.4 × 3.4 mm; TR = 25 ms; TE = 419 ms; Flip = 80°; 145 frames).
2. First-level Processing
  The preprocessing of resting-state fMRI consisted of correcting for motion and time-slice acquisition, brain extraction, spatial smoothing with 5 mm FWHM isotropic Gaussian kernel, high-pass temporal filtering (100 s), nonlinear registration of structural images to a standard Montreal Neurological Institute (MNI) template (2 × 2 × 2 mm), boundary-based registration (BBR) to high-resolution T1 images. In addition, we also performed motion scrubbing [27] and regressed motion parameters, cerebrospinal fluid (CSF), white matter, the global brain signal, and their 1st derivatives [28]. Quality control criteria for each BOLD run were as follows: 1) no motion scrubbing greater than 50% of the run; and 2) no frame movement larger than 1 functional voxel. CAPS scores were not correlated with head motion in the scanner during the fMRI [relative motion: *r*_(64)_ = − 0.0497, *p* = 0.6989; absolute motion: *r*_(64)_ = − 0.1232, *p* = 0.3361] or dMRI [translation motion: *r*_(60)_ = 0.0445; *p* = 0.7355; rotation motion: *r*_(60)_ = 0.1324; *p* = 0.309] scans.
3. Network Construction
  Using a meta-analytically derived functional brain atlas from Power et al. (which we refer to throughout as the functional atlas), we partitioned the brain into 264 cortical and subcortical ROIs [29]. To construct a DMN-specific network—and in order to avoid a potential bias in selecting the DMN component(s) post-signal decomposition—we decided to adopt a validated and reliable DMN mask established by Yeo et al. [30]. Of the original 264 ROIs, 64 ROIs were found to belong to the DMN map and were subsequently used for the analyses. We extracted and averaged time series from all voxels within each ROI. We then generated pairwise Pearson correlation coefficients from each ROI and proceeded to apply a Fischer z-transformation (Fz) to stabilize the correlation coefficient variance, resulting in a DMN-specific 64 × 64 Fz matrix. Each of the two 5-min runs were processed separately until this point and then averaged prior to further analyses. Since it has been shown that regressing the global BOLD signal may induce anticorrelations, the interpretation of which remains unclear [31], [32], negative weights were initially discarded from analyses. However, the analyses were in part repeated with the complete networks (where both positive and negative weights are retained), on a post hoc basis to ensure consistency. Since certain graph theoretical measures such as the clustering coefficient require that weights fall between 0 and 1 [18], Fz matrices were then rescaled by maximal weight (Fz_scaled_ = Fz/max(abs(Fz))); between 0 and 1 for the positive-only networks, and between − 1 and 1 for the full networks.
  Concerns have been raised with regards to the reliability of weighted networks, namely that they are prone to noise [33], [34]. To ascertain that our main results were not driven by this influence, we attempted two alternative approaches. In the first approach, we used stringent thresholding criteria (retaining the top 15% of the edges with the strongest weight) to attenuate the effect of such false positives. The second approach avoids such an arbitrary threshold and uses statistical significance to determine the presence of edges between nodes [33]. In addition to having the advantage of sparsifying the network in a principled way, such an approach may also reduce false negatives compared to highly stringent (arbitrary) cutoffs [33].
  All analyses that follow were applied to the weighted matrices, using the weighted-variants of the functions included in the Brain Connectivity Toolbox (https://sites.google.com/site/bctnet) [12], [35], GenLouvain [36] (http://netwiki.amath.unc.edu/GenLouvain), and the Community Detection Toolbox (http://commdetect.weebly.com), under MATLAB 2016b (MathWorks Inc., Massachusetts, United States). Brain maps were generated using BrainNet Viewer (https://www.nitrc.org/projects/bnv) [37].
4. Connectivity Strength, Integration, and Segregation
  As a primary outcome measure, we used the overall connectivity strength (*S*). We first calculated the nodal strength—the weighted variant of nodal degree, calculated as the sum of all neighboring link weights. When averaged across the network, it provides information about the overall connectivity strength or wiring investment of the network [12]. We calculated two secondary global measures to further characterize information transfer across the network: global efficiency and clustering coefficient. Global efficiency measures network integration, the efficiency of overall network communication across the network. Mathematically, it is inversely related to the distance between nodes (i.e., the number of edges separating them, taking into account the weight of these edges) [15]. The clustering coefficient measures network segregation by quantifying local interconnectivity. It is mathematically related to the number and weight of triangles formed by nodes and edges [17], [18]. While the clustering coefficient is calculated on a nodal basis, an average across all nodes can be calculated and interpreted as a measure of overall network segregation—the capacity of specialized processing. All subsequent mentions of the clustering coefficient refer to the mean clustering coefficient across all nodes. Weighted generalizations of the clustering coefficient [18], and the global efficiency [12], [15], were adopted for the calculations.

### 2.3 Accounting for connectedness

**Fig. 1:**
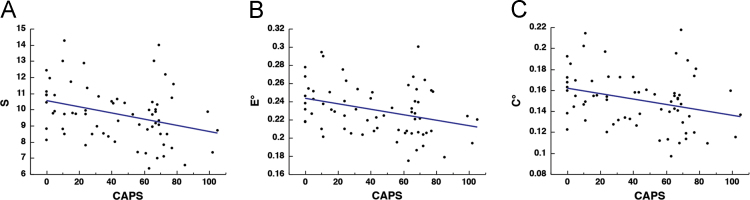
Relationship between CAPS and non-normalized metrics. Scatter plots depicting the correlation between PTSD severity, as measured by the Clinician Administered PTSD Scale (CAPS) and *S* [*r*_(64)_ = − 0.329, *p* = 0.0075] (A), *E*^*o*^ [*r*_(64)_ = − 0.299, *p* = 0.0157] (B), and *C*^*o*^ [*r*_(64)_ = − 0.296, *p* = 0.0168] (C).

Our results revealed that strength (*S*), non-normalized global efficiency (*E*^*o*^) and clustering coefficient (*C*^*o*^), were all negatively associated with CAPS [*S*: *r*_(64)_ = − 0.329, *p* = 0.0075; *E*^*o*^: *r*_(64)_ = − 0.299, *p* = 0.0157; *C*^*o*^: *r*_(64)_ = − 0.296, *p* = 0.0168] (Fig. 1).

It is expected that a biologically-relevant varying level of connectedness exists across participants, here captured using *S*. However, the changes in global efficiency and clustering coefficient may be an artifactual consequence of changes in *S*. in order to enable a meaningful comparative analysis of integration and segregation measures across participants, the difference in node connectedness needs to be accounted for [38], [39]. To ensure that the higher order network metrics of were not an artifact driven by different level of connectedness, we attempted several strategies based on normalization with null model networks [12], [38], [40], [41]. The results are summarized in Table 2.

**Table 2:** Summary of results of the different methods to account for connectedness in order to obtain a measurement of global efficiency and clustering coefficient.

	**Null model performance**			**Resulting normalized measures**	
	*Correlation between Random and original network strength (stronger is better)*	*Correlation between Random and original network density (stronger is better)*	*Correlation between normalized strength and CAPS (weaker is better)*	*Correlation between variants of the global efficiency and CAPS*	*Correlation between variants of the clustering coefficient and CAPS*
**Non-normalized**	N/A	N/A	N/A	*E*^*o*^	*C*^*o*^
				*r* = − 0.299	*r* = − 0.296
				*p* = 0.0157	*p* = 0.0168
**Phase normalized**	*r* = 0.083	*r* = 0.090	*r* = − 0.344	*E*^*norm_phase*^	*C*^*norm_phase*^
	*p* = 0.5087	*p* = 0.7102	*p* = 0.0050	*r* = − 0.301	*r* = − 0.313
				*p* = 0.0149	*p* = 0.0113
**Phase normalized – matched strength (but not density)**	*r* = 1.000	*r* = 0.019	*r* = − 0.239	*E*^*norm_phase_match_S*^	*C*^*norm_phase_match_S*^
	*p* < 0.0001*	*p* = 0.8837	*p* = 0.048	*r* = − 0.265	*r* = − 0.230
				*p* = 0.0360	*p* = 0.0701
**Phase normalized – matched density (but not strength)**	*r* = 0.080	*r* = 1.000	*r* = − 0.320	*E*^*norm_phase_match_D*^	*C*^*norm_phase_match_D*^
	*p* = 0.5245	*p* < 0.0001*	*p* = 0.0095	*r* = − 0.300	*r* = − 0.330
				*p* = 0.0150	*p* = 0.0073
Rewired (preserving strength and density)**	*r* = 1.000	*r* = 1.000	*r* = 0.0597	*E*^*norm_rewire*^	*C*^*norm_rewire*^
	*p* < 0.0001*	*p* < 0.0001*	*p* = 0.6365*	*r* = − 0.299	*r* = 0.334
				*p* = 0.0157	*p* = 0.0065
**Covarying for *S* at the level of statistical analysis**	N/A	N/A	N/A	*E*^*o_partial*^	*C*^*o_partial*^
				*r* = − 0.062	*r* = 0.191
				*p* = 0.6255	*p* = 0.1278

* Denotes satisfactory performance on benchmark.

** Denotes entry with satisfactory performance on all benchmarks.

For each participant, the global efficiency and mean clustering coefficient were calculated for the original and a corresponding random surrogate networks, and the normalized variants of these measures which were used in the statistical analysis were defined as *E*^*norm*^ = *E*^*o*^/*E*^*rand*^ and *C*^*norm*^ = *C*^*o*^/*C*^*rand*^, respectively [12]. *E*^*rand*^ and *C*^*rand*^ were calculated as the mean global efficiency and mean overall clustering coefficient values for these metrics over the 100 random surrogates. Linear regressions were used to examine the relationship between CAPS scores and the DMN topological measures. In both cases, we conducted regressions with *S*^*norm*^ (*S*^*norm*^ = *S*/*S*^*rand*^) and CAPS to verify that the effect of weight has been mitigated.

We used two different methods to build random surrogates: phase randomization and rewiring [35], [42], [43], [44], and tested the ability of each to mitigate the effect of individual differences in varying level of connectivity.

**Fig. 2:**
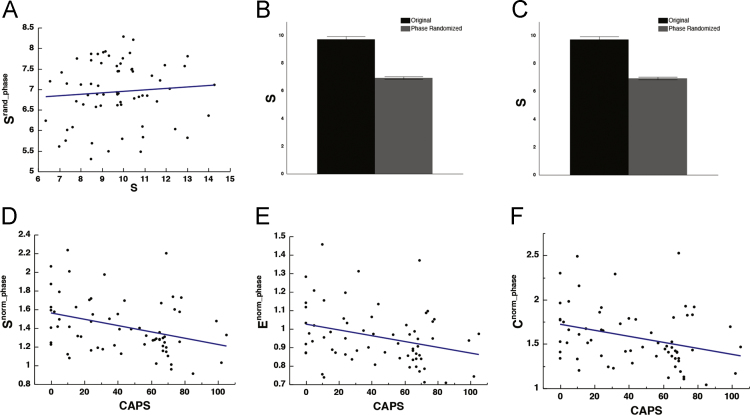
The effect of phase randomization. Scatter plot depicting the correlation between *S* and *S*^*norm_phase*^ [*r*_(64)_ = 0.083, *p* = 0.5087] (A). Bar graphs depicting means and standard error of strength and binary density in original [mean strength = 9.72 ± 0.22; mean density = 0.71 ± 0.0068] (B) and random surrogate networks [mean strength = 6.94 ± 0.093; mean density = 0.50 ± 0.0013] (C). Scatter plots depicting the correlation between CAPS and *S*^*norm_phase*^ [*r*_(64)_ = − 0.344, *p* = 0.0050], *E*^*norm_phase*^ [*r*_(64)_ = − 0.301, *p* = 0.0149], and *C*^*norm_phase*^ [*r*_(64)_ = − 0.313, *p* = 0.0113] (D-F).

For phase randomization, we started with the BOLD time series, applied a Fourier transform, scrambled the phase and inverted the transform, as described in [42], [43]. The resultant null model networks were not matched to the originals in terms of strength or degree, and the procedure did not mitigate the effect of varying level of connectedness on CAPS (Fig. 2).

**Fig. 3:**
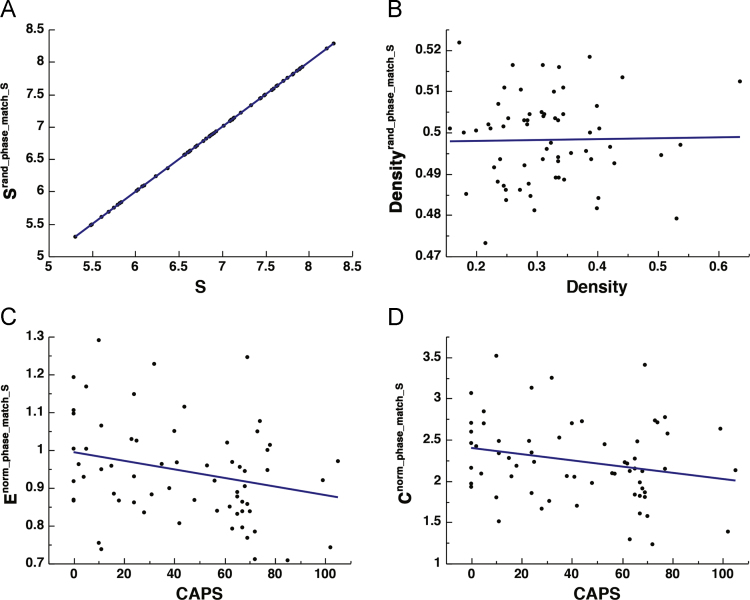
The effect thresholding original-surrogate pairs to match the strength. Scatter plot depicting the correlation between: *S* and *S*^*rand_phase*^ [*r*_(64)_ = 1.000, *p* < 0.0001] (A), binary density of the original and random surrogates [*r*_(64)_ = 0.019, *p* = 0.8837] (B), CAPS and *E*^*norm_phase*^ [*r*_(64)_ = − 0.265, *p* = 0.0360] (C), and CAPS and *C*^*norm_phase*^ [*r*_(64)_ = − 0.230, *p* = 0.0701] (D).

**Fig. 4:**
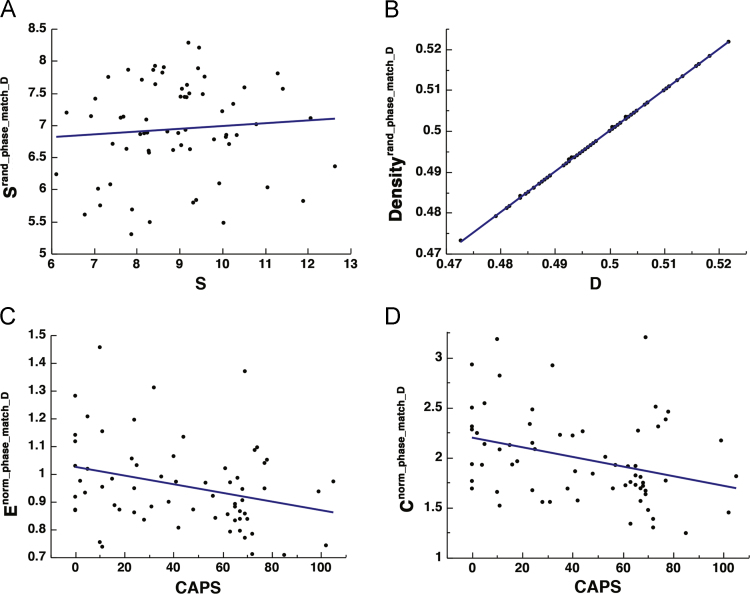
The effect thresholding original-surrogate pairs to match the binary density. Scatter plot depicting the correlation between: *S* and *S*^*rand_phase*^ [*r*_(64)_ = 0.080, *p* = 0.5245] (A), binary density of the original and random surrogates [*r*_(64)_ = 1.000, *p* < 0.0001] (B), CAPS and *E*^*norm_phase*^ [*r*_(64)_ = − 0.300, *p* = 0.0150] (C), and CAPS and *C*^*norm_phase*^ [*r*_(64)_ = − 0.330, *p* = 0.0073] (D).

Thresholding strategies have been proposed to enhance the matching of the random surrogates to the original networks in terms of edge connectedness [43]. Such methods are typically applied to binary networks, where matching by binary density or degree is sufficient. Here we used proportional thresholding to match network pairs for either degree or strength. In each case, the lowest degree and strength were determined for each original-random pair, and a threshold was used to sparsify the network with the higher degree or strength to ensure matching. This resulted in networks that were either matched with respect to degree or strength, but not both. Further, the patterns found in the non-normalized global efficiency and clustering coefficient metrics were preserved. See Figs. 3 and 4.

While, proportional thresholding of random networks may successfully ensure matching in binary networks, this method did not appear to do so successfully in our weighted networks; further, thresholding has been shown to have a direct effect on graph metrics [38].

**Fig. 5:**
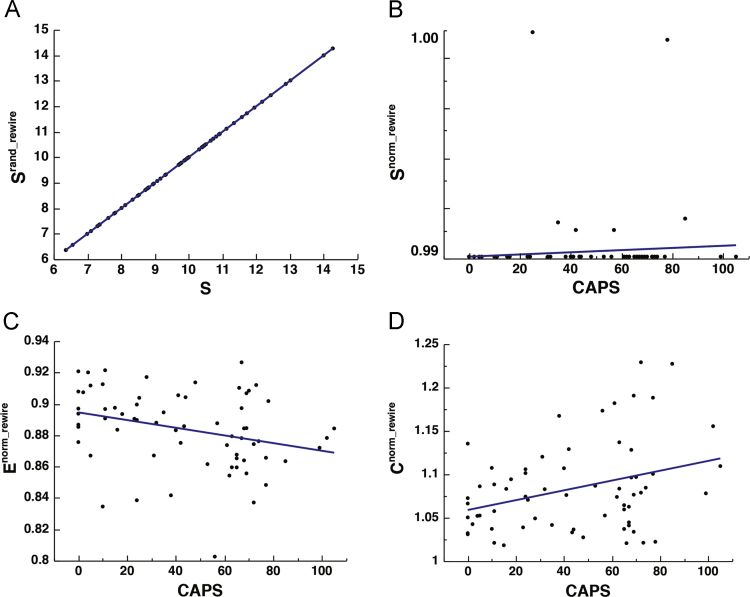
The effect of rewiring randomization. Scatter plots depicting the correlation between: *S* and *S*^*norm_rewire*^ [*r*_(64)_ = 1.000, *p* < 0.0001] (A), CAPS and *S*^*norm_rewire*^ [*r*_(64)_ = 0.0597, *p* = 0.6365], *E*^*norm_rewire*^ [*r*_(64)_ = − 0.299, *p* = 0.0157], and *C*^*norm_rewire*^ [*r*_(64)_ = 0.334, *p* = 0.0065] (B-D).

For rewiring, edges of the original networks were randomized (number of edge swaps = 10, weight sorting frequency = 1) [35], [44], resulting in a null model network with a preserved degree and strength distribution [strength and binary density matched between original and surrogates: *r* = 1.000, *p* < 0.0001]. Recent concerns have been raised regarding the adequacy of null models based on rewiring algorithms when used with correlation-based networks [43]. Correlation-based networks (such as those derived from fMRI/BOLD) have an intrinsic transitive nature that is lost after random rewiring [43]. The implication being that this will result in artificially inflated small-world properties, due to transitive qualities in the original but not randomized networks. However, despite their inadequacy to represent the intrinsic small-world properties of individual networks (e.g., when used to make empirical observation in observed network organization vs. random organization), they may still be useful for the cross-subject comparative purposes when the desired effect is attenuating the effect of connectivity difference, and have indeed been used as such [40], [45]. This process successfully mitigated the effect of varying level of connectedness on CAPS (Fig. 5).

**Fig. 6:**
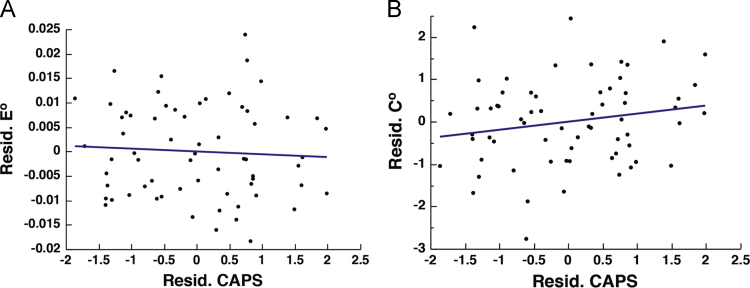
The effect of controlling for *S* at the level of statistical analysis. Scatter plots depicting the correlation between PTSD severity, as measured by the Clinician Administered PTSD Scale (CAPS), and DMN integration and segregation. (A-B) represent the *E*^*o*^*, C*^*o*^, and CAPS residuals after controlling for *S* [*E*^*o_residual*^: *r*_(62)_ = − 0.062, *p* = 0.6255; *C*^*o_residual*^: *r*_(62)_ = 0.191, *p* = 0.1278].

Finally, attempts were made to correct for this varying level of connectivity at the level of statistical analysis by conducting regressions between CAPS and *E*^*o*^ and *C*^*o*^ while covarying for *S*, although this approach has been known to be strict and may discard true higher-order topological properties [40]. See Fig. 6.

### 2.4 Alternate edge definition

Our first-level processing pipeline included regressing the global BOLD signal—a procedure known to induce artificial anticorrelations in constructed functional correlation-based networks. The interpretability of these anticorrelations remains uncertain [31], [32]. Due to these factors, we discarded negative weights from the main analyses. Here we repeat the analyses in part to assess whether similar results would be found with the full signed (positive and negative) networks. Here also total strength (*S*) was calculated as the sum of all neighboring link weights (positive and negative), and the mean calculated across the network. For the clustering coefficient, we adopted a generalization for signed networks that takes both positive and negative weights into account simultaneously [46]. The global efficiency was calculated by using absolute value of the weights.

The construction of networks from time series based on Pearson correlations are prone to false positive connections [33], [34]. To assess the extent to which our main results were influenced by this noise, we attempted two approaches: in the first one, we used stringent thresholding criteria (retaining the top 15% of the edges with the strongest weight) to attenuate the effect of such false positives.

The second approach avoids such an arbitrary threshold and uses statistical significance to determine the presence of edges between nodes [33]. In addition to having the advantage of sparsifying the network in a principled way, such an approach may also reduce false negatives compared to highly stringent (arbitrary) cutoffs [33]. Here we follow the analytic (extremum) method described in [33], while controlling for false discovery rate (FDR) with *q* = 0.001 [33], [47]. This yielded networks with a mean density of 0.55 (range = 0.45,0.61).

The results are presented in Table 3.

**Table 3:** Alternate edge definition.

	**S**		**E^norm_rewire^**		**C^norm_rewire^**	
	**r**	**p**	**r**	**p**	**r**	**p**
**Positive weighted (analysis in the main NeuroImage article****[51]**)	− 0.329	0.0075	− 0.299	0.0157	0.334	0.0065
**Signed weighted**	− 0.339	0.0058	− 0.159	0.2066	0.327	0.0078
**Thresholded (edge density = 15%)**	− 0.279	0.0245	− 0.228	0.0681	0.197	0.1164
**Networks with statistical significance****[33]****(weighted thresholded)**	− 0.373	0.0022	− 0.054	0.667	0.252	0.0429
**Networks with statistical significance****[33]****(binarized)**	− 0.303	0.0142	− 0.049	0.699	0.275	0.0267

### 2.5 Alternate parcellation atlas

In order to assess the robustness of our network-wide findings and ascertain that the detected changes were not due the functionally-derived brain parcellation atlas, the analysis was repeated using a novel multi-modal parcellation of the cerebral cortex by Glasser et al. adopted by the Human Connectome Project (HCP) [48]. Briefly, under a volume-based version of this parcellation scheme, the DMN map was found to comprise 83 brain parcels.

Consistent with the primary analysis using the functional parcellation atlas, the same pattern of associations between increased PTSD symptom severity and *S* [*r*_(64)_ = − 0.299, *p* = 0.0146], *E*^*norm_rewire*^ [*r*_(64)_ = − 0.364, *p* = 0.0026], and *C*^*norm_rewire*^ [*r*_(64)_ = 0.276, *p* = 0.0248] were found.

### 2.6 Alternate DMN definitions

For the primary analyses, the decision to avoid a sample-specific DMN extraction using decomposition methods was motivated by several factors. 1) The potential utility of the measures as biomarkers relies on sample-independence and easy replicability across studies; 2) it is known that the spatial extent of different ICNs is altered by numerous factors—notably, psychopathology. We wanted to capture this alteration statistically using connectivity strength, rather than spatial extent. Carrying out the analysis on DMN extracted from a mixed sample of non-PTSD combat-exposed and PTSD participants may result in a reduced sensitivity when assessing DMN connectivity strength; 3) given than functional imaging modalities are noise-prone, ICNs decomposed from small samples may yield idiosyncratic results; 4) for the purpose of unifying definitions and consistency across neuropsychiatric disorders, it is reasonable to adopt an established ICN map identified from a large healthy sample.

From the available ICN maps, the one identified by Yeo et al. [30] is the most widely used, derived from a large sample of healthy individuals (*N* = 1,000), and most importantly, is atlas-independent, i.e., it can be adopted irrespective of the ROI parcellation scheme (e.g., functional or multi-modal parcellation in our case).

However, in order to verify that the results we obtained with the primary analysis were not due to an intricate property of the adopted DMN definition, we repeated the analyses with a sample-specific DMN decomposition, and with an alternate established DMN definition [29]. To identify a sample-specific DMN definition, we applied a multi-iterative generalization of the Louvain community detection algorithm on a sample-mean network (using the same parcellation atlas as the primary analysis; total number of nodes = 264) [12], [29], [49]. At *γ* = 2, we obtained a non-fragmented component with 67 nodes with the highest spatial overlap with the primary DMN consensus map. The alternate consensus DMN definition was adopted from Power et al. [29] (and makes use of the same functional parcellation atlas; under this definition, the DMN consists of 58 nodes).

In both cases, the pattern of results was similar to the primary analyses. Sample-specific DMN: [*S*: *r*_(64)_ = − 0.278, *p* = 0.0256; *E*^*norm_rewire*^: *r*_(64)_ = − 0.207, *p* = 0.0976; *C*^*norm_rewire*^: *r*_(64)_ = 0.289, *p* = 0.0162]. Alternate established DMN: [*S*: *r*_(64)_ = − 0.271, *p* = 0.0312; *E*^*norm_rewire*^: *r*_(64)_ = − 0.159, *p* = 0.2120; *C*^*norm_rewire*^: *r*_(64)_ = 0.249, *p* = 0.0446].

### 2.7 Assessing for putative confounds

To assess the effect of putative confounds, we conducted partial correlations between CAPS and the DMN measures, controlling for each of the following variables: age, sex, BDI, BAI, CES, TBI, alcohol or substance use disorder status, psychoactive medication status, WTAR, education, and handedness. Since our sample consisted exclusively of combat-exposed individuals, the interaction between CES and CAPS was also explored. Subgroup analysis excluding subjects taking psychotropic medications and with psychiatric comorbidities were also conducted. Data can be found in Table 4.

**Table 4:** CAPS correlations with DMN network characteristics.

**Covariate**	***S***	***E***^***norm_rewire***^	***C***^***norm_rewire***^	***n***
None	− 0.329	− 0.299	0.334	65
Age	− 0.334	− 0.299	0.338	65
Sex	− 0.325	− 0.293	0.328	65
Age & Sex	− 0.331	− 0.294	0.332	65
WTAR	− 0.337	− 0.303	0.338	61
Education	− 0.307	− 0.284	0.310	62
CES	− 0.331	− 0.261	0.346	60
BDI	− 0.235* (0.06)	− 0.217* (0.08)	0.171* (0.18)	65
BAI	− 0.299	− 0.274	0.223* (0.08)	65
Medication	− 0.300	− 0.232* (0.06)	0.283	65
Mild TBI	− 0.333	− 0.269* (0.07)	0.312	46
SUD	− 0.271	− 0.229* (0.08)	0.275	59
Handedness	− 0.318	− 0.283	0.319	65
**Subgroup**	***S***	***E***^***norm_rewire***^	***C***^***norm_rewire***^	***n***
Exc. medications	− 0.488	− 0.343	0.430	41
Exc. comorbidities	− 0.221* (0.394)	− 0.239* (0.356)	0.282* (0.273)	17

Values are the correlations coefficients between CAPS and each of the DMN connectivity measures, covarying for potential confounds or in subgroups. All correlations were statistically significant with *p* < 0.05, except where *p* is otherwise noted in parenthesis (denoted by *). The number of observations for each entry is provided. Abbreviations – CAPS: Clinician Administered PTSD Scale for the DSM-IV; *S*: connectivity strength; *E*^*norm_rewire*^: global efficiency normalized with rewired random surrogates; *C*^*norm_rewire*^: clustering coefficient normalized with rewired random surrogates; WTAR: Wechsler Test of Adult Reading; CES: Combat Exposure Scale; BDI: Beck Depression Inventory; BAI: Beck Anxiety Inventory; TBI: Traumatic Brain Injury; SUD: Substance/Alcohol Use Disorder.

A subgroup analysis excluding subjects taking psychotropic medications revealed the same pattern across all measures [*S*: *r*_(40)_ = − 0.488, *p* = 0.0012; *E: r*_(40)_ = − 0.343, *p* = 0.028; *C*: *r*_(40)_ = 0.430, *p* = 0.005]. Another subgroup was attempted with subjects with no psychiatric comorbidities (*n* = 17). No statistically significant relationships were found, but directions of the correlations were preserved: [*S*: *r*_(16)_ = − 0.221; *E: r*_(16)_ = − 0.239; *C*: *r*_(16)_ = 0.282, all *p* > 0.3].

### 2.8 Group comparison

While we opted for a single group dimensional analysis for our primary analyses, here we compare the global measures between PTSD and non-PTSD subjects. This analysis was conducted to ensure the robustness of the results beyond the correlation analyses, and to enable more meaningful comparisons between our results and those of in other studies that adopt a group approach.

To this aim, we divided our sample into two groups: those who met DSM-IV criteria for PTSD (PTSD; *n* = 35) and those who did not, i.e., combat exposed controls (CC; *n* = 30), and general linear models were used for the statistical analysis. Group characteristics can be found in Table 5.

**Table 5:** Group characteristics.

	**CC (Mean ± *SEM* or %)**	**PTSD (Mean ± *SEM* or %)**	**p value**
**N**	30	35	N/A
**Age (years)**	34.3 ± 1.9	35.2 ± 1.6	0.7
**Sex (% female)**	3/27	4/31	0.9
**WTAR Standard Score**	102.3 ± 1.6	104.5 ± 1.3	0.3
**Education (years)**	14.5 ± 0.3	13.6 ± 0.3	0.06
**CAPS**	15.9 ± 2.8	66.9 ± 2.7	< 0.001*
**CES**	14.1 ± 1.5	20.9 ± 1.8	0.007*
**BDI**	11.8 ± 1.8	25.5 ± 1.7	< 0.001*
**BAI**	7.1 ± 1.1	18.2 ± 1.7	< 0.001*
**MDD**	7/16	3/24	0.09
**SUD**	3/24	9/23	0.1
**Anxiety Disorder**	0/24	4/26	0.06
**Psychotropic Medication**	5/25	19/16	0.002*
**Mild TBI**	12/8	17/9	0.7
**Handedness (% left)**	3/27	8/27	0.2

Abbreviations – SEM: Standard Error of Means; WTAR: Wechsler Test of Adult Reading; CAPS: Clinician Administered PTSD Scale for the DSM-IV; CES: Combat Exposure Scale; BDI: Beck Depression Inventory; BAI: Beck Anxiety Inventory; PTSD: Posttraumatic Stress Disorder; MDD: Major Depressive Disorder; SUD: Substance/Alcohol Use Disorder; Anxiety: Panic Disorder, Generalized Anxiety Disorder, Obsessive Compulsive Disorder; TBI: Traumatic Brain Injury.

* *p* < 0.05.

**Fig. 7:**
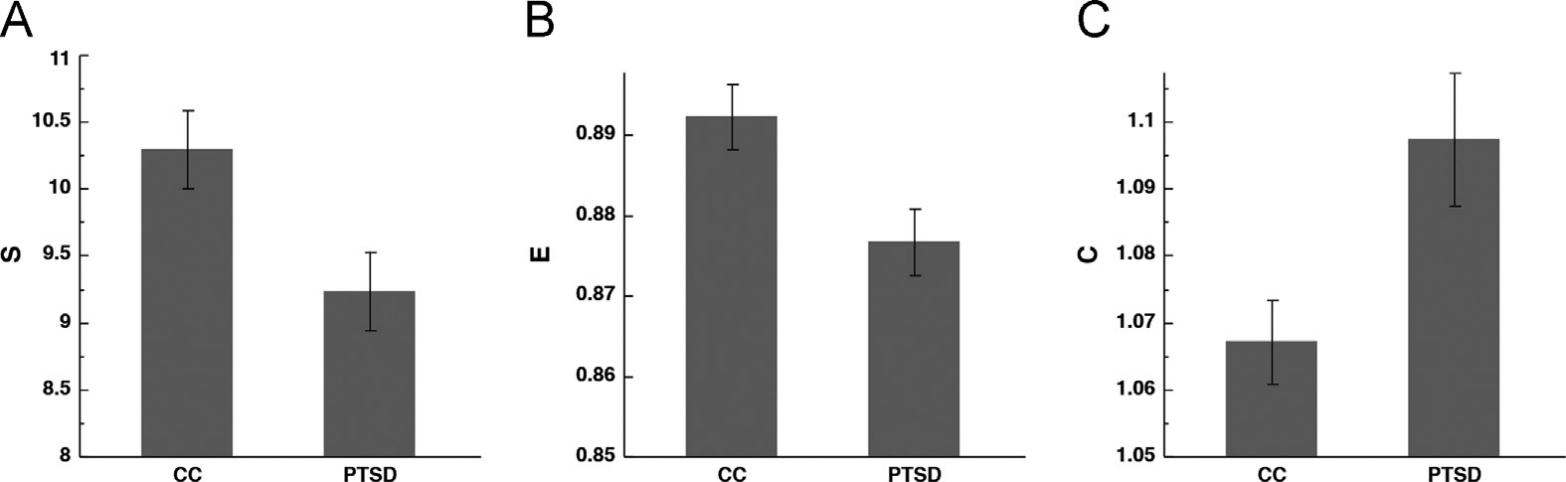
Group comparisons of DMN characteristics. Bar graphs depicting means and standard error of *S*, *E*, and *C* across the CC and PTSD groups. *S* [CC: *mean* = 10.30 ± 0.31; PTSD: *mean* = 9.23 ± 0.28; *p* = 0.014, *df* = 64], *E* [CC: *mean* = 0.892 ± 0.004; PTSD: *mean* = 0.877 ± 0.004; *p* = 0.01, *df* = 64], and *C* [CC: *mean* = 1.067 ± 0.009; PTSD: *mean* = 1.097 ± 0.008; *p* = 0.016, *df* = 64].

Consistent with the dimensional analysis, there was a statistically significant difference between the two groups across the 3 assessed global measures, namely, that in PTSD compared to CC, there׳s lower *S*, lower *E*, but higher *C*. See Fig. 7 and Table 6.

**Table 6:** Group comparisons with DMN network characteristics.

	**S (Mean ± SEM)**			**E^norm_rewire^ (Mean ± SEM)**			**C^norm_rewire^ (Mean ± SEM)**			**n**
**Subgroup**	CC	PTSD	*p*	CC	PTSD	*p*	CC	PTSD	*p*	
All	10.30 ± 0.31	9.23 ± 0.28	0.014	0.892 ± 0.004	0.877 ± 0.004	0.01	1.067 ± 0.009	1.097 ± 0.008	0.016	65
Exc. medications	10.537 ± 0.322	8.938 ± 0.402	0.004	0.894 ± 0.004	0.880 ± 0.006	0.069	1.064 ± 0.009	1.093 ± 0.011	0.045	41
Exc. comorbidities	9.526 ± 0.407	8.932 ± 0.383	0.305	0.885 ± 0.011	0.870 ± 0.011	0.344	1.079 ± 0.014	1.104 ± 0.014	0.232	17
All – covarying for CES, BDI, BAI, medications and TBI	10.522 ± 0.544	8.913 ± 0.423	0.047	0.892 ± 0.008	0.876 ± 0.006	0.178	1.069 ± 0.015	1.093 ± 0.12	0.300	42

Abbreviations – SEM: Standard Error of Means; CES: Combat Exposure Scale; BDI: Beck Depression Inventory; BAI: Beck Anxiety Inventory; TBI: Traumatic Brain Injury.

Subgroup analyses were conducted for individuals who were not taking psychotropic medications (*n* = 41), and for individuals without psychiatric comorbidities (*n* = 17). Numerical relationships largely held, although statistical significance was not always present. Results can be found in Table 6.

### 2.9 Non-ICN restricted global efficiency and overall strength

One of the goals of our contribution was to establish an ICN-restricted approach to the study of psychopathology, notably for practical use in biomarker development. Such approach discards connections exterior to the ICN that is being investigated. This limitation is particularly salient for the calculation of global efficiency, which is based on the shortest path lengths. For example, the shortest path length between 2 nodes in the DMN may not be entirely within the DMN. However, we made the implicit assumption that an ICN-restricted weighted calculation—where only paths that are part of the ICN are included in the networks—would approximate all important (i.e., high-weight) paths, by virtue of lower between-ICN connectivity compared to within-ICN.

To ascertain the validity of this assumption, we attempted an alternate calculation of DMN efficiency that is not ICN-restricted in paths. Here, whole-brain networks were constructed (264 nodes), in an analogous fashion to the DMN networks. The connection-length matrices necessary for the global efficiency calculation were calculated between all nodes—irrespective of ICNs. The mean of the inverse shortest path length for nodes belonging to the DMN was calculated and normalized using the rewiring-based null model. A similar pattern compared to the ICN-restricted global efficiency was found when a linear regression with CAPS was attempted [*r*_(64)_ = − 0.321, *p* = 0.0092].

Similarly, nodal strength was calculated as the sum of all neighboring link weights—irrespective of ICNs and averaged across the nodes belonging to the DMN. Here, the non-ICN restricted DMN strength was not found to be significantly correlated with CAPS [*r*_(64)_ = − 0.103, *p* = 0.4154], indicating that in PTSD, the disturbance is mainly driven by changes within the DMN.

### 2.10 Edge statistical testing

Results of network-based statistical testing (connection threshold *t* = 3.5; permutations = 10,000; corrected *α* < 0.05) and edge-wise FDR (permutations = 100,000; corrected *α* < 0.05) revealed subnetworks with edges that are weakened with increasing PTSD symptom severity (Table 7) [50].

**Table 7:** Edges associated with increased PTSD severity.

**Edge**		
**Node 1**	**Node 2**	**Test Stat.**
Right Angular Gyrus (S096)	Left Inferior Orbitofrontal (S137)	− 3.90*
Left Anterior Cingulum (S111)	Left Inferior Orbitofrontal (S137)	− 4.29*
Left Anterior Cingulum (S113)	Left Inferior Orbitofrontal (S137)	− 3.56
Left Superior Frontomedial (S115)	Left Inferior Orbitofrontal (S137)	− 3.75
Left Superior Frontomedial (S115)	Right Supramarginal Gyrus (S204)	− 4.47*
Left Middle Temporal Gyrus (S084)	Left Inferior Orbitofrontal (S137)	− 3.56

* Denotes edges that were also statistically significant with FDR in place of NBS as the method to control for family-wise error. Labels are based on Automated Anatomical Labeling (AAL) atlas. “S[---]” in parenthesis denote the index labels given in the original Power et al. atlas, and were included for reference.

To increase statistical power and increase sensitivity, NBS leverages the extent to which abnormal edges are interconnected [50]. Therefore, this method results in one or more *connected* components of significantly differing edges. To ascertain that the identified edges are not idiosyncratic as a result of this bias, we used a confirmatory approach with FDR as the link-based controlling method for family-wise error, although this method is known to be less sensitive [50]. Again, the DMN connectivity matrices of each participant were entered as dependent variables and the total CAPS score as predictor variable (permutations = 100,000; corrected *α* < 0.05) [50].
